# Patients characteristics and outcomes of prolonged mechanical ventilation after critical illness transferred to a weaning unit

**DOI:** 10.1186/2197-425X-3-S1-A318

**Published:** 2015-10-01

**Authors:** J Rubio, JA Rubio Mateo-Sidron, E Palma Gonzalez, R Sierra Camerino, F Carmona Espinazo, F Fuentes Morillas

**Affiliations:** Hospital Puerta del Mar, ICU, Cádiz, Spain; Hospital Infanta Cristina, Badajoz, Spain; Hospital Puerta del Mar, Cádiz, Spain

## Introduction

Recent trends seem to point out that growing numbers of patients requiring prolonged mechanical ventilation (PMV) and protracted weaning after critical illness with eventually account for a large portion of all Intensive Care Unit (ICU) cost and bed occupancy. Specialized Weaning Unit (WU), may facilitate weaning through focused multidisciplinary expert team, but due to considerable variability of interventions and organization outcomes remain unclear.

## Objectives

To characterize patients, risk factors and outcomes of patients who require tracheostomy, PMV and weaning after critical illness and are transferred to a WU.

## Methods

A retrospective, descriptive study of tracheostomized and clinically stable patients who required PMV (> 21 days) and weaning (> 7 days) admitted to a 23-bed university intensive care unit and were transferred to a WU over a 4-year period (2011-2014) after critical illness. Demographics characteristics, clinical status at admission, reasons for needing PMV, complications and clinical status at discharge were recorded and analysed from clinical charts and electronic databases. Basic descriptive statistics are presented, mean ± SD, median (interquartile range), and percentages.

## Results

Twenty-six patients were included in the study. Most were male (57.7%). Age 60,88 ± 16.5 years. APACHE II score 27.5 (12 - 46). The most frequent admission category was medical (61.5%), and the main diagnostics groups were neurological 9 (34,6%) and respiratory (15,4%). ICU and WU length of stay were 45.5 (11 - 88) and 74.5 (3 - 244) days respectively, with an in-hospital mortality rate of 15.4%. The main reasons for mechanical ventilation in WU were neuromuscular illness (73%), and were on mechanical ventilation for 84 (4 - 533) days. Seven-teen (65.4%) patients were discharged to home, 76.5% of them could be decannulated and 35% were out with mechanical ventilation. Four (15.4%) patients were transferred to other long-care facilities.

## Conclusions

This specific WU care resulted in low mortality rates, high success of weaning and considerable home discharge rates of ICU patients with prolonged mechanical ventilation and weaning.
